# Childhood visual impairment causes and barriers to accessing eye care: A suggested approach for Africa

**DOI:** 10.4102/phcfm.v16i1.4556

**Published:** 2024-07-16

**Authors:** Saif H. Alrasheed, Zoelfigar D. Mohamed, Muhammed S. Alluwimi

**Affiliations:** 1 Department of Optometry, College of Applied Medical Sciences, Qassim University, Buraydah, Saudi Arabia; 2 Department of Binocular Vision, Faculty of Optometry and Visual Sciences, Al-Neelain University, Khartoum, Sudan; 3 Department of Optometry, College of Health Sciences, University of Buraimi, Al Buraimi, Oman

**Keywords:** global health, incidence, prognosis, affordability, school, child

## Abstract

**Background:**

Childhood vision impairment (VI) has a significantly harmful effect on both health and social outcomes.

**Aim:**

To assess the causes of childhood VI, to determine obstacles to accessing eye care services and to develop a strategy for the childhood eye care system in African nations.

**Method:**

This systematic review was conducted by searching several online databases, including; Scopus, PubMed, ProQuest, Web of Science, Google Scholar, Ebsco and Medline. They focussed on articles available between 2003 and 2023. These studies were conducted to evaluate the causes of childhood VI and to assess obstacles to accessing eye care services in African countries.

**Results:**

The main causes of childhood VI in African nations can be avoided with timely diagnosis and an appropriate management strategy. The leading obstacles to accessing childhood eye care services were a lack of availability, accessibility and affordability. In addition to these barriers, we found that there are concerns with quality of services, primary health care system, geographic barriers, incorrect health beliefs, inappropriate parental perception, a lack of knowledge, attitudes and inadequate practices related to paediatric eye care.

**Conclusion:**

The main causes of childhood VI were uncorrected refractive error (RE), amblyopia, cataract and corneal opacities that can be avoided with timely diagnosis and an appropriate management strategy. While the main obstacles to accessing childhood eye care services were a lack of availability, accessibility, affordability and healthcare system.

**Contribution:**

The recommended strategy for childhood eye-care services includes models for delivery and training.

## Introduction

Childhood visual impairment (VI) can significantly impact child development, negatively affecting their quality of life.^1,2^ The World Health Organization (WHO) reported that good vision plays a major role in the growth of children and teenagers.^3^ Globally, the prevalence of childhood VI varies depending on socioeconomic status, ranging from low in affluent areas to high in extremely poor ones.^4,5,6,7^ The results of Yekta et al.’s systematic review in 2022 indicated that the global prevalence of childhood VI, based on uncorrected visual acuity (UCVA), was 7.26%, in which the African region reported the highest prevalence of childhood blindness.^8^ Several studies reported that the prevalence of VI in school-aged children in various African countries is noteworthy; for example, a study in South Africa reported a prevalence rate of 2.15%,^9^ while another study in Sudan found a prevalence rate of 5.5%.^10^ In addition, a study in Ethiopia found a prevalence rate of 9.5%.^11^

The main causes of childhood VI vary significantly and are influenced by factors like socioeconomic status and access to basic eye care services.^12,13,14^ However, almost 80% of these causes are avoidable with early diagnosis and treatment.^5,6,7,8,9,10,11,12,13,14,15,16^ Uncorrected refractive errors (REs) were the leading cause of childhood VI, followed by amblyopia and congenital cataract.^8,16^

Identifying affected children within the community is crucial because it impacts on the demand for services and the effectiveness of school programme screening. Early detection of affected children helps reduce the global burden of childhood VI and enhances public health outcomes.^17,18^ Regular check-ups, along with access to primary, secondary and tertiary eye care, are important for the prevention of avoidable childhood blindness and VI among school-age children.^13^

Obstacles to access eye care services for children are wide-ranging and encompass factors that hinder children from obtaining these services, ultimately leading to negative consequences for both service distribution and access.^19^ Unfortunately, eye care services for the children in many African nations lack infrastructure, equipment, resources and qualified professionals.

The current workforce on the African continent is insufficient, with most countries falling short of the WHO recommended ratio of eye care professionals to population.^20,21^ Furthermore, the primary obstacles to obtaining eye care services for young children in Africa are cost and unavailability.^22^ Thus, the current systematic review was performed to assess the causes of childhood VI and to determine obstacles to accessing eye care in order to develop an approach for the childhood eye care system in African countries.

## Methods

### Search plan and selection criteria

The current review was performed in compliance with the guidelines established by PRISMA 2020.^23^ The authors conducted a comprehensive search of several online databases, such as PubMed, Web of Science, ProQuest, Scopus, Medline and Google Scholar for studies published between 2003 and 2023. The articles included in this systematic review were evaluated to assess the causes of VI in children and to determine barriers to the availability and accessibility of childhood eye care.

**FIGURE 1:**
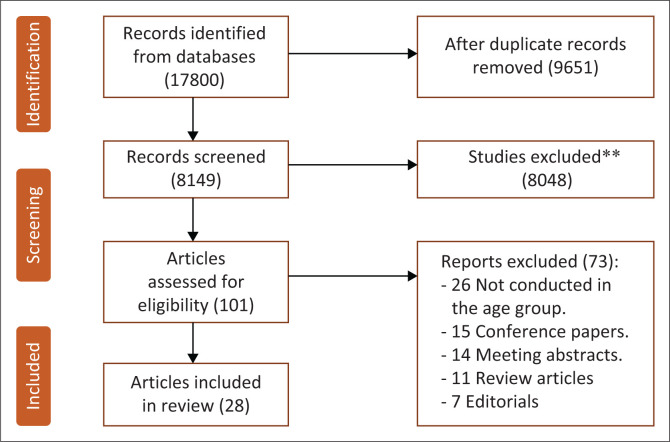
Identification of studies used in the present study.

The search keywords were ‘Vision impairment in children’ or ‘childhood vision impairment in African nations’, and ‘Causes of childhood visual impairment’ or ‘Barriers to Eye care services’ in African countries. This review was limited to papers published in English journals that underwent a peer-review process. Previous research has focussed on understanding the reasons behind childhood VI and the accessibility obstacles to childhood eye care in Africa.^24,25,26,27,28,29,30,31,32,33,34,35,36,37,38,39,40,41,42,43,44,45,46,47,48,49^ This study, however, only considered studies that utilised various methodologies and excepted editorial discussions, conference papers, meeting abstracts and studies that lacked important information such as causes and barriers to access childhood eye care as shown in Figure 1.

### Data extraction

The authors gathered crucial information, including the main author, year of publication, nation of the study, participant’s characteristics (age and sample size), the causes of childhood VI and barriers to access the childhood eye care services. This information is organised and presented in Table 1 and Table 2.

**TABLE 1:** The main causes of childhood vision impairment in Africa.

First name year	Country	Sample size	Age	Main causes (%)				
				RE	Corneal opacities	Retinal	Cataract	Amblyopia
Ekpenyong et al. 2020^24^	Nigeria	2110	6–17	70.70	1.50	0.50	0.50	3.00
Ebri et al. 2019^25^	Nigeria	4241	10–18	91.30	NA	1.20	0.30	4.10
Alrasheed et al. 2016^26^	Sudan	1678	6–15	57.00	0.90	13.10	3.70	5.60
Mohamed et al. 2017^27^	Sudan	1062	5–15	36.00	21.00	3.60	21.50	NA
Bezabih et al. 2017^28^	Ethiopia	718	6–18	70.27	8.11	5.41	NA	2.70
Kedir et al. 2014^29^	Ethiopia	570	7–15	54.00	8.10	10.80	2.70	5.40
Darge et al. 2017^30^	Ethiopia	378	6–15	77.30	NA	NA	4.50	4.50
Abdi et al. 2020^31^	Somaliland	1204	6–15	76.80	0.60	NA	0.60	22.00
Kumah et al. 2013^32^	Ghana	2435	12–15	71.70	4.60	5.90	NA	9.90
Naidoo et al. 2003^33^	South Africa	4890	5–15	63.60	3.70	9.90	NA	7.30
Muma et al. 2020^16^	Kenya	3240	5–16	62.00	NA	NA	NA	24.00
Nallasamy et al. 2011^34^	Botswana	241	≤ 15	33.00	NA	NA	31.00	31.00
Gyawali et al. 2017^35^	Eritrea	22 509	< 16	12.10	15.70	NA	19.70	12.10

**Total**	**-**	**40 145**	**-**	**59.67**	**7.13**	**6.30**	**9.40**	**11.00**

Note: Please see the full reference list of the article, Alrasheed SH, Mohamed ZD, Alluwimi MS. Childhood visual impairment causes and barriers to accessing eye care: A suggested approach for Africa. Afr J Prm Health Care Fam Med. 2024;16(1), a4556. https://doi.org/10.4102/phcfm.v16i1.4556, for more information.

RE, refractive error; NA, not available.

**TABLE 2:** Common Obstacles to accessing childhood eye care services in African states.

First name year	Country	Sample size	Barriers to accessing childhood eye care services in African countries
Agarwal et al. 2010^36^	Three African countries	27 health tertiary facilities	Accessibility, affordability and availability
Sukati et al. 2018^37^	Swaziland	173 parents	Absence of knowledge about childhood eye disorders and care services
Ugalahi et al. 2020^38^	Nigeria	164 children	Cost of childhood eye care facilities
Chan et al. 2017^39^	Tanzania	1051 contributors	Parents attitudes towards childhood eye conditions
Alrasheed et al. 2018^40^	Sudan	18 eye care professionals	Accessibility, affordability and availability
Wanyama 2013^41^	Kenya	125 paediatricians	Awareness and practices of childhood eye disorders among paediatricians
Belaynew et al. 2014^42^	Ethiopia	1315 families	Awareness and practices of the community towards childhood VI
Kotb et al. 2010^43^	Egypt	100 children	Attitudes and awareness towards eye disorders
Schulze et al. 2014^44^	Malawi	58 parents	Awareness and practices towards childhood eye care services
Kumah et al. 2017^45^	Ghana	100 parents	Awareness and practices of childhood VI
Chan, et al. 2020^46^	South Africa	93 Children	Attitude and perceptions towards eye care services
Sukati, et al. 2019^47^	Swaziland	15 eye care professionals	Accessibility and availability of childhood eye care services
Alrasheed et al. 2018^48^	Sudan	434 participants	Attitudes and perceptions towards RE treatment
Ugalahi et al. 2020^38^	Nigeria	164 children	Affordability to eye care services
Oguego et al. 2018^49^	Nigeria	833 Pupils	Awareness and eye care misconceptions

**Total**	**-**	**4670**	**-**

Note: Please see the full reference list of the article, Alrasheed SH, Mohamed ZD, Alluwimi MS. Childhood visual impairment causes and barriers to accessing eye care: A suggested approach for Africa. Afr J Prm Health Care Fam Med. 2024;16(1), a4556. https://doi.org/10.4102/phcfm.v16i1.4556, for more information.

RE, refractive error; VI, vision impairment.

## Results

### Characteristics of the included studies

In total, 17 800 articles were identified by the authors (depicted in Figure 1). Following the elimination of duplicate headings, 8149 articles were examined. After scanning the summaries and abstracts of these 8149 articles, 8011 articles that failed to satisfy the inclusion criteria were removed. Furthermore, 73 articles were eliminated from consideration after reviewing their full transcripts, as they were found to be lacking essential information. The final filtration included 28 articles from different African countries (Table 1 and Table 2). The articles included in the present study were published between 2003 and 2023. The studies involved a total sample size of 40 145 children and assessed the main causes of VI. In addition, the total number of participants that assessed the barriers of accessing childhood eye care services was 4670, which varied among schoolchildren, parents and eye care professionals.

### The main causes of childhood vision impairment in Africa

Table 1 displays the factors contributing to childhood VI in Africa. A total of 13 reviewed articles revealed that the leading causes of childhood VI in African countries were uncorrected refractive error (RE) (59.67%), amblyopia (11.00%), cataract (9.40%), corneal opacities (7.13%) and retinal disorders (6.30%). The uncorrected RE was the primary cause of childhood VI in Africa, with percentages ranging from 91.3% in Nigeria^25^ to 12.1% in Eritrea.^35^ Amblyopia was the second cause of VI with highest prevalence found in Botswana at 31.0%,^34^ followed by corneal opacities which was the most prevalent in Eritrea at 15.7%,^35^ and cataracts which was the highest in Sudan at 21.5%.^27^

### Common barriers to accessing childhood eye care services in African countries

The remaining 15 reviewed articles showed that the core barriers were the awareness, practices and attitudes of the community towards childhood VI^41,42,43,44,45,48^ followed by lack of affordability accessibility, availability of eye care services for children. Furthermore, paediatric eye care was deemed unimportant in numerous African care systems.^36,38,40,47^ Studies reviewed showed that there were other factors hampering the utilisation of eye care for children which include lack of knowledge about childhood eye disorders and care services, parents’ attitudes towards childhood eye conditions, geographic blockades, demographic and socio-economic conditions^36,37,38,39,40,41,42,43,44,45,46,47,48,49^ (Table 2).

### Childhood eye care systems: A recommended approach for the African region

Regarding the above-mentioned findings, the recommended method for a childhood eye care system should address the following areas:

### Vision screening for school-aged children

The reviewed studies^24,25,26,27,28,29,30,31,32,33,34,35^ conducted among African children showed a high prevalence of RE, eye disease, that led to childhood VI. Based on these findings, we suggest that schoolteachers should be trained for vision screening one per class at the school level. The referral criteria for children with VI are a visual acuity of 6/12 or worse. Children who fail vision screening should be referred to nearby primary health centres at the community level, where at least one optometrist is available. These children should undergo comprehensive eye examinations. Children with uncorrected RE should receive optical correction through public–private partnerships or hospital-run initiatives. Children with complicated eye conditions like strabismus, low vision or other eye problems should be referred to local hospitals that have specialist eye care providers. Whereas children with more complex eye diseases such as cataracts, corneal opacities and retinal disorders should be referred to district hospitals with paediatric eye care providers for further treatment.

### Eliminating the common obstacles to accessing eye care services for children

Findings of research^37,39,41,42,43,44,45^ showed that the knowledge, attitudes and practices related to eye conditions among parents and children were poor. The present study suggests the following eye care approach: To raise awareness about childhood eye disorders, health promotion materials should be created and distributed in clinics and public locations. These materials should inform the community about the signs, symptoms and consequences of untreated eye diseases, emphasise the importance of regular examinations and early detection, highlight the consequences of delayed treatment and provide information on where to seek eye care. Additionally, eye care providers should deliver health education programmes through TV and radio broadcasts to dispel misconceptions about childhood eye care and increase awareness of the impact of uncorrected RE and childhood VI.

The findings^36,37,38,39,40,41,42,43,44,45,46,47,48,49^ revealed that misconceptions among children and their parents regarding the effectiveness of spectacles as a treatment for RE. Furthermore, the community holds the belief that traditional medicine is the most effective approach for treating childhood eye diseases and RE. Therefore, we propose that eye care professionals should educate patients during routine eye examinations about the benefits of wearing spectacles and the significance of using them to correct RE. It is crucial to emphasise the effectiveness of patching therapy and early treatment for childhood eye disorders. To address misconceptions about spectacles, education programmes through mass media, schools and public presentations by eye care providers and psychologists should be implemented. Additionally, training traditional healers to screen and refer patients to clinics would help integrate them into the healthcare system.

Most of the African population fall below the poverty line. Studies^36,38,40^ showed that the cost of eye care services is the leading obstacle for many people in African states. To address these challenges, we recommend securing financial support from the government, private sector and non-government organisations (NGOs) to support childhood eye care services in African states. Implementing cost-effective community-based screening programmes is essential, and the government should consider offering special tax exemptions to ensure the affordability of childhood eye care services and optical corrections.

### Human resources development

The findings of the reviewed studies^36,37,38,39,40,41,42,43,44,45,46,47,48,49^ showed the lack of primary eye care services and RE screening programmes for primary schoolchildren; furthermore, there is an absence of paediatric eye care professionals in African countries. In response to these findings, we suggest that training personnel to meet the necessities of paediatric optometrists should be developed and implemented at the local institutions. Additionally, community leaders, parents and teachers should be trained on how to screen visual disorders by monitoring visual behaviours or symptoms that they may notice.

The Ministry of Health in African nations should train enough paediatric optometrists, general practitioners, ophthalmologists, low vision specialists and binocular vision specialists at the primary, secondary and tertiary levels.

The reviewed studies^36,40,41,42^ revealed the disparity and inappropriate distribution of eye care professionals in most African countries. This situation worsens because of insufficient skills among eye care personals. The recommended solution is for African Ministries of Health to review and adjust the eye care provider distribution system, considering both distance to services and population density. Additionally, annual training courses should be implemented to enhance the knowledge and skills of eye care professionals, with support and funding from Ministries of Health, the private sector and NGOs.

### Infrastructure

Studies^36,40,41,47^ have identified insufficient basic instruments for paediatric eye examinations and infrastructure as the primary obstacles to accessing childhood eye care services in African countries. The authors recommend that the government, private sector and donors collaborate to provide essential ophthalmic instruments and mobile clinic units. Additionally, the present study proposes that the government and other organisations supply low-cost or free optical and low-vision devices to support visually impaired children.

### Policy changes

The present study found that eye care services are not part of primary health care in certain African countries,^36,40,41^ and health insurance does not cover such services. Therefore, there is a need to re-evaluate the public health system, including the implementation of health insurance that covers eye care services and the integration of eye care into primary health care. Moreover, African nations should establish government policies concerning the availability of spectacles, contact lenses, affordable diagnostic eye drops and the inclusion of eye care in the health insurance system.

### Partnerships

In numerous African countries, there is insufficient collaboration among eye health care providers (both public and private), stakeholders and policy makers regarding childhood eye care.^36,37,38,39,40,41,42,43,44,45,46,47,48,49^ To improve the sustainability and effectiveness of childhood eye care, it is crucial to establish multi-collaboration initiatives involving the Ministry of Health, Ministry of Education, public and private eye care providers, NGOs, faith organisations, community leaders and local institutions.

## Discussion

According to global estimates, approximately 1.4 million children are blind, with three-quarters of them residing in African countries.^22^ Our reviewed studies^24,25,26,27,28,29,30,31,32,33,34,35^ showed that the main causes of childhood VI were uncorrected RE, amblyopia, cataract and corneal opacities that can be avoided with timely diagnosis and an appropriate management strategy. These findings are consistent with the global report of the prevalence and causes of VI in children^8^ as well as Indian and Nepalese findings.^50,51^

Furthermore, our findings revealed that the leading obstacles to accessing childhood eye care services were lack of affordability, accessibility, availability and insufficient healthcare professionals.^52^ The reviewed studies^36,37,38,39,40,41,42,43,44,45,46,47,48,49^ found additional factors influencing the utilisation of childhood eye services, including geographic obstacles, health views, parental perception, lack of knowledge, attitudes and inadequate paediatric practices. Environmental factors and socioeconomic status were also found to negatively impact the accessibility of eye care services in African nations.

In African states, accessing paediatric eye care is impeded by factors such as low quality of paediatric practice, limitations in the health insurance system and inadequate distribution of healthcare professionals.^36,37,38,39,40,41,42,43,44,45,46,47,48,49^ These barriers need to be addressed by reconfiguring the public health system and implementing comprehensive vision screening programmes in primary schools to detect and treat childhood eye diseases and uncorrected RE at early stages. The accessibility for the eye care in urban areas is mostly developed in many African states. For example, health care services are mostly located in urban parts of Nigeria, leaving rural parts without access to services.^21^ Similarly in Sudan, most of the eye care services are situated in the central and northern parts of the country, leaving many regions in the south and west without health care services.^40^ Borrel et al.^19^ highlighted that the unequal distribution of eye care professionals in Africa creates substantial obstacles to accessing eye services. For example, in Cameroon, 80% of eye care providers are concentrated in just 2 out of 10 provinces. Similarly, in Ethiopia, most eye care providers are in the capital city, whereas in Sudan, 70% of health facilities are centralised in the capital city Khartoum. A study conducted in Ethiopia revealed that the primary obstacles to accessing eye services were associated with the cost of services, aligning with the aforementioned factors.^53^ Findings from a survey conducted in South Africa indicated that 80.5% of patients received the suggested management; however, affordability issues prevented 36.4% of individuals from accessing the treatment.^54^

The main obstacle to providing child eye care in many African nations is the absence of eye care services. To overcome this obstacle, it is essential for the government and donors to support the basic eye care services, as they play a crucial role in eliminating these barriers. Providing assistance to low-income families in accessing good quality of eye care services in public hospitals would enable the early diagnosis and management of childhood eye diseases. In most African nations, where most of the populace lives below the poverty line, parents cannot afford eye services for their children. The Ministry of Health in African countries and donors should allocate financial support for training paediatric eye care professionals and school-teaching staff. This training would enable the detection of eye problems through annual vision examinations in schools, leading to timely referrals for visually impaired children. Initially, teachers should conduct school vision screenings with one teacher per class. Children with vision equal to or less than 6/12 should be referred to the primary health centres for comprehensive eye examinations, while RE services should be provided at the community level. Children with low vision, strabismus and other eye conditions should be referred to local hospitals with paediatric eye care specialists. Those with complex eye diseases should be referred to paediatric ophthalmologists at district hospitals.

In most African countries, the existing health infrastructure is inadequate, and there is a significant absence of childhood eye services and skilled eye care providers. These challenges require immediate attention and solutions from both the government and NGOs focussed on avoidable childhood VI. Borrel et al.^19^ highlighted that eye health care for children in numerous African states is a significant concern but not given importance on the public health programme.

Implementing a school vision screening programme could be the most effective approach to identifying uncorrected RE and other causes of childhood VI across African countries. It is crucial to provide training to eye care providers in order to recognise signs of childhood eye disorders, enabling them to refer children with VI for proper diagnosis, treatment and management. World Health Organization emphasised the importance of establishing child eye care system in the community.^6^ To accomplish this, health workers at the national, and local levels should work together with partners from various sectors, including healthcare providers, NGOs, academics, private sector and the media, to facilitate the implementation of an improved children health care system.

In many African countries, there is an absence of information on childhood eye diseases, VI, barriers to accessing eye care and published plans for childhood eye care. The study recommends that the Ministry of Health, the private sector and NGOs should provide support and funding for conducting research on childhood eye diseases, VI and barriers to access. This would enhance the quality of research on childhood eye care and the evidence generated can be utilised to develop and implement effective strategies. Additionally, the government should encourage local universities and institutions to conduct research on emerging childhood eye priorities and effective diagnostic and treatment methods.

## Conclusion

**FIGURE 2:**
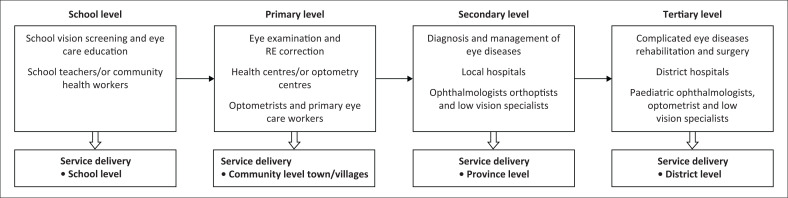
Suggested model for paediatric eye care service delivery for African states.

**FIGURE 3:**
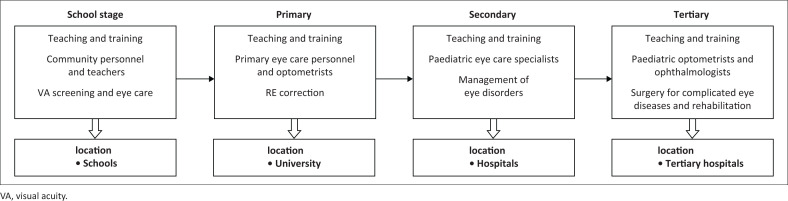
Model for paediatric eye care training needs for African nations.

The main causes of childhood VI in African states were uncorrected RE, amblyopia, cataract and corneal opacities that could be avoided with timely diagnosis and an appropriate management strategy. Whereas, the main obstacles to accessing eye care services were a lack of availability, accessibility and affordability, in addition to concerns with the primary care system, geographic obstacles, health beliefs, parental perception, lack of knowledge, attitudes and inadequate practices related to eye care. Therefore, the suggested childhood eye care plan for African nations should address several aspects of eye care system. This approach can be assessed and adapted according to the countries and the situation of childhood eye care system. Thus, the present study recommends that the strategy for childhood eye-care services should include models for delivery (Figure 2) and training (Figure 3).

## References

[1] DeCarlo DK, McGwin Jr G, Bixler ML, Wallander J, Owsley C. Impact of paediatric vision impairment on daily life: Results of focus groups. Optometry Vis Sci. 2012;89(9):1409–1416. 10.1097/OPX.0b013e318264f1dc PMC3474353 22863790

[2] Castañeda YS, Cheng-Patel CS, Leske DA, et al. Quality of life and functional vision concerns of children with cataracts and their parents. Eye. 2016;30(9):1251–1259. 10.1038/eye.2016.134 27391939 PMC5023803

[3] World Health Organization (WHO). Blindness and vision impairment. World Health Organization; 2023 [cited 2024 Feb 07]. Available from: https://www.who.int/news-room/fact-sheets/detail/blindness-and-visual-impairment

[4] Solebo AL, Rahi J. Epidemiology, aetiology and management of visual impairment in children. Arch Dis Childhood. 2014;99:375–379. 10.1136/archdischild-2012-303002 24148891

[5] Zelalem M, Abebe Y, Adamu Y, Getinet T. Prevalence of visual impairment among school children in three primary schools of Sekela Woreda, Amhara regional state, north-west Ethiopia. SAGE Open Med. 2019;7:2050312119849769. 10.1177/2050312119849769 31205693 PMC6537079

[6] Gilbert C, Foster A. Childhood blindness in the context of vision 2020: The right to sight. Bull World Health Organ. 2001;79(3):227–232. 11285667 PMC2566382

[7] Kong L, Fry M, Al-Samarraie M, Gilbert C, Steinkuller PG. An update on progress and the changing epidemiology of causes of childhood blindness worldwide. J Am Assoc Paediatr Ophthalmol Strabismus. 2012;16(6):501–507. 10.1016/j.jaapos.2012.09.004 23237744

[8] Yekta A, Hooshmand E, Saatchi M, et al. Global prevalence and causes of visual impairment and blindness in children: A systematic review and meta-analysis. J Curr Ophthalmol. 2022;34(1):1–15. 10.4103/joco.joco_135_21 35620376 PMC9128433

[9] Naidoo KS, Raghunandan A, Mashige KP, et al. Refractive error and visual impairment in African children in South Africa. Invest Ophthalmol Vis Sci. 2003;44(9):3764–3770. 10.1167/iovs.03-0283 12939289

[10] Zeidan Z, Hashim K, Muhit MA, Gilbert C. Prevalence and causes of childhood blindness in camps for displaced persons in Khartoum: Results of a household survey. East Mediterr Health J. 2007;13(3):580–585. 17687831

[11] Mehari ZA, Yimer AW. Prevalence of refractive errors among schoolchildren in rural central Ethiopia. Clin Exp Optom. 2013;96(1):65–69. 10.1111/j.1444-0938.2012.00762.x 22784031

[12] Solebo AL, Teoh L, Rahi J. Epidemiology of blindness in children. Arch Dis Childhood. 2017;102(9):853–857. 10.1136/archdischild-2016-310532 28465303

[13] Mohamed ZD, Abdu M, Alrasheed SH. Management plan for childhood visual impairment in traditional Quranic boarding schools in Al-Gazira state of Sudan. Albasar Int J Ophthalmol. 2018;5(1):1–5. 10.4103/bijo.bijo_8_18

[14] Alrasheed SH, Naidoo KS, Clarke-Farr PC. Childhood eye care services in South Darfur State of Sudan: Learner and parent perspectives. Afr Vision Eye Health. 2016;75(1):a315. 10.4102/aveh.v75i1.315 PMC6244194 30456975

[15] Resnikoff S, Pascolini D, Etya’Ale D, et al. Global data on visual impairment in the year 2002. Bull World Health Organ. 2004;82(11):844–851. 15640920 PMC2623053

[16] Muma S, Obonyo S. The prevalence and causes of visual impairment among children in Kenya–the Kenya eye study. BMC Ophthalmol. 2020;20:1–5. 10.1186/s12886-020-01665-w 33028254 PMC7542701

[17] Sharma A, Congdon N, Patel M, Gilbert C. School-based approaches to the correction of refractive error in children. Survey Ophthalmol. 2012;57(3):272–283. 10.1016/j.survophthal.2011.11.002 22398336

[18] Muhit MA, Shahjahan M, Hassan A, Wazed A, Ahmed N. Parental knowledge, attitude and practice related to blindness of children in some selected Upazilla of Bangladesh. Mymensingh Med J. 2011;20:671–679. 22081188

[19] Borrel A, Dabideen R, Mekonen Y, Overland L. Child eye health in Africa: The status and way forward. Cape Town: African Child Policy Forum, ORBIS Africa; 2013, p. 1–37.

[20] Balasubramaniam SM, Kumar DS, Kumaran SE, Ramani KK. Factors affecting eye care–seeking behaviour of parents for their children. Optometry Vis Sci. 2013;90(10):1138–1142. 10.1097/OPX.0000000000000010 24037060

[21] Ntsoane MD, Oduntan OA. A review of factors influencing the utilization of eye care services. Afr Vision Eye Health. 2010;69(4):182–192. 10.4102/aveh.v69i4.143

[22] Alrasheed SH. A systemic review of barriers to accessing paediatric eye care services in African countries. Afr Health Sci. 2021;21(4):1887–1897. 10.4314/ahs.v21i4.47 35283961 PMC8889803

[23] Page MJ, McKenzie JE, Bossuyt PM, et al. The PRISMA 2020 statement: An updated guideline for reporting systematic reviews. Int J Surg. 2021;88:18. 10.1016/j.ijsu.2021.105906 33789826

[24] Ekpenyong BN, Naidoo K, Ndep A, Akpan M, Ekanem E. Prevalence, and determinants of visual impairment amongst school-aged children in Southern Nigeria. Afr Vision Eye Health. 2020;79(1):a534. 10.4102/aveh.v79i1.534

[25] Ebri AE, Govender P, Naidoo KS. Prevalence of vision impairment and refractive error in school learners in Calabar, Nigeria. Afr Vision Eye Health. 2019;78(1):a487. 10.4102/aveh.v78i1.487

[26] Alrasheed SH, Naidoo KS, Clarke-Farr PC. Prevalence of visual impairment and refractive error in schoolaged children in South Darfur State of Sudan. Afr Vision Eye Health. 2016;75(1):a355. 10.4102/aveh.v75i1.355

[27] Mohamed ZD, Binnawi KH, Abdu M. Prevalence and causes of childhood blindness and visual impairment in Quranic boarding schools in Al-Gazira state of Sudan. Sudanese J Ophthalmol 2017;9(2):44–49. 10.4103/sjopthal.sjopthal_1_18

[28] Bezabih L, Abebe TW, Fite RO. Prevalence and factors associated with childhood visual impairment in Ethiopia. Clin Ophthalmol. 2017;2017:1941–1948. 10.2147/OPTH.S135011 PMC5685140 29184383

[29] Kedir J, Girma A. Prevalence of refractive error and visual impairment among rural school-age children of Goro District, Gurage Zone, Ethiopia. Ethiopian J Health Sci. 2014;24(4):353–358. 10.4314/ejhs.v24i4.11 PMC4248035 25489200

[30] Darge HF, Shibru G, Mulugeta A, Dagnachew YM. The prevalence of visual acuity impairment among school children at Arada Subcity primary schools in Addis Ababa, Ethiopia. J Ophthalmol. 2017;17(1):1–5. 10.1155/2017/9326108 PMC5494567 28706737

[31] Abdi Ahmed Z, Alrasheed SH, Alghamdi W. Prevalence of refractive error and visual impairment among school-age children of Hargesia, Somaliland, Somalia. East Mediterr Health J. 2020;26(11):1362–1370. 10.26719/emhj.20.077 33226104

[32] Kumah BD, Ebri A, Abdul-Kabir M, et al. Refractive error, and visual impairment in private school children in Ghana. Optometry Vis Sci. 2013;90(12):1456–1461. 10.1097/OPX.0000000000000099 24270594

[33] Naidoo KS, Raghunandan A, Mashige KP, et al. Refractive error and visual impairment in African children in South Africa. Invest Ophthalmol Vis Sci. 2003;44(9):3764–3770. 10.1167/iovs.03-0283 12939289

[34] Nallasamy S, Anninger WV, Quinn GE, Kroener B, Zetola NM, Nkomazana O. Survey of childhood blindness and visual impairment in Botswana. Br J Ophthalmol. 2011;1–5. 10.1136/bjo.2010.189068 21242581 PMC4127423

[35] Gyawali R, Bhayal BK, Adhikary R, Shrestha A, Sah RP. Retrospective data on causes of childhood vision impairment in Eritrea. BMC Ophthalmol. 2017;17(1):1–8. 10.1186/s12886-017-0609-x 29166895 PMC5700735

[36] Agarwal PK, Bowman R, Courtright P. Child eye health tertiary facilities in Africa. J Am Assoc Paediatr Ophthalmol Strabismus. 2010;14(3):263–266. 10.1016/j.jaapos.2010.02.007 20603061

[37] Sukati VN, Moodley VR, Mashige KP. Knowledge and practices of parents about child eye health care in the public sector in Swaziland. Afr J Prim Health Care Fam Med. 2018;10(1):a1808. 10.4102/phcfm.v10i1.1808 PMC6244139 30456970

[38] Ugalahi MO, Olusanya BA, Fagbemi OO, Baiyeroju AM. Delays in uptake of surgery for childhood cataract at a child eye health tertiary facility in sub-Saharan Africa. Eur J Ophthalmol. 2020;30(2):280–283. 10.1177/1120672119827770 30747005

[39] Chan VF, Minto H, Mashayo E, Naidoo KS. Improving eye health using a child-to-child approach in Bariadi, Tanzania. Afr Vision Eye Health. 2017;76(1):a406. 10.4102/aveh.v76i1.406

[40] Alrasheed SH, Naidoo KS, Clarke-Farr PC, Binnawi KH. Building consensus for the development of child eye care services in South Darfur State of Sudan using the Delphi technique. Afr J Prim Health Care Fam Med. 2018;10(1):e1–e9. 10.4102/phcfm.v10i1.1767 PMC6244194 30456975

[41] Wanyama SP. Knowledge, attitude, and practice of eye diseases in children among pediatricians in Kenya. Doctoral dissertation. University of Nairobi.

[42] Belaynew WT, Berihun MZ, Tadesse AA, Yared AW. Knowledge and practice on childhood blindness among communities in Northwest Ethiopia: Implications to blindness prevention programs. JOECSA. 2014;17(2):50–58.

[43] Kotb SA, Gadallah MA, Marzouk SA. Self-esteem and quality of life among visually impaired children in Assiut City, Egypt. J Am Sci. 2011;7(8):47–57.

[44] Schulze Schwering M, Finger RP, Barrows J, Nyrenda M, Kalua K. Barriers to uptake of free pediatric cataract surgery in Malawi. Ophthalmic Epidemiol. 2014;21(3):138–143. 10.3109/09286586.2014.892139 24597953

[45] Kumah BD, Abdulkabir M, Kobia-Acquah E, Amponsah MA, Ablordeppey RK. Knowledge of childhood blindness among mothers visiting a children’s Hospital in the Kumasi Metropolis, Ghana. Adv Ophthalmol Vis Syst. 2017;7(3):00221. 10.15406/aovs.2017.07.0022

[46] Chan VF, Singer S, Naidoo KS. Disability-related-distress in primary school learners with vision impairment because of uncorrected refractive error in KwaZulu-Natal Province, South Africa – A qualitative study. PLoS One. 2020;15(3):e0229108. 10.4102/aveh.v78i1.47 32126114 PMC7053722

[47] Sukati V, Moodley VR, Mashige KP. Knowledge and practices of eye health professionals about the availability and accessibility of child eye care services in the public sector in Swaziland. Afr Vision Eye Health. 2019;78(1), a471. 10.4102/aveh.v78i1.471

[48] Alrasheed SH, Naidoo KS, Clarke-Farr PC. Attitudes and perceptions of Sudanese high-school students and their parents towards spectacle wear. Afr Vision Eye Health. 2018;77(1):a392. 10.4102/aveh.v77i1.392

[49] Oguego N, Okoye OI, Okoye O, et al. Eye health myths, misconceptions, and facts: Results of a cross-sectional survey among Nigerian school children. Fam Med Prim Care Rev. 2018;20(2):144–148. 10.5114/fmpcr.2018.76458

[50] Wadhwani M, Vashist P, Singh SS, Gupta V, Gupta N, Saxena R. Prevalence and causes of childhood blindness in India: A systematic review. Indian J Ophthalmol. 2020;68(2):311–315. 10.4103/ijo.IJO_2076_18 31957718 PMC7003592

[51] Bhattarai S. The scenario of childhood blindness and its remedy in Nepal. J Patan Acad Health Sci. 2021;8(2):120–125. 10.3126/jpahs.v8i2.31126

[52] Adugna MB, Nabbouh F, Shehata S, Ghahari S. Barriers, and facilitators to healthcare access for children with disabilities in low and middle income sub-Saharan African countries: A scoping review. BMC Health Serv Res. 2020;20:15. 10.1186/s12913-019-4822-6 31906927 PMC6945633

[53] Melese M, Alemayehu W, Friedlander E, Courtright P. Indirect costs associated with accessing eye care services as a barrier to service use in Ethiopia. Trop Med Int Health. 2004;9(3):426–431. 10.1111/j.1365-3156.2004.01205.x 14996373

[54] Mashige KP, Martin C. Utilization of eye care services by elderly persons in the northern Ethekwini district of Kwa-Zulu-Natal province, South Africa. Afr Vision Eye Health. 2011;70(4):175–181. 10.4102/aveh.v70i4.113

